# Pandemic (H1N1) 2009 Virus and Down Syndrome Patients

**DOI:** 10.3201/eid1608.091931

**Published:** 2010-08

**Authors:** Rogelio Pérez-Padilla, Rosario Fernández, Cecilia García-Sancho, Francisco Franco-Marina, Octavio Aburto, Hugo López-Gatell, Ietza Bojórquez

**Affiliations:** National Institute of Respiratory Diseases, Mexico City, Mexico (R. Pérez-Padilla, R. Fernández, C. García-Sancho, F. Franco-Marina, O. Aburto); Secretariat of Health, Mexico City (H. López-Gatell, I. Bojórquez)

**Keywords:** Running title: Pandemic (H1N1) 2009 and Down Syndrome, influenza, Down syndrome, viruses, pandemic (H1N1) 2009, dispatch

## Abstract

We compared prevalence of hospitalization, endotracheal intubation, and death among case-patients with and without Down syndrome during pandemic (H1N1) 2009 in Mexico. Likelihoods of hospitalization, intubation, and death were 16-fold, 8-fold, and 335-fold greater, respectively, for patients with Down syndrome. Vaccination and early antiviral drug treatment are recommended during such epidemics.

Down syndrome is the most common chromosomal abnormality in persons worldwide; prevalence is ≈1/1,000 live births (*1*). It is characterized by a variety of dysmorphic features, congenital malformations, and other health problems. Several risk factors for influenza also occur in Down syndrome patients (*2*): congenital heart disease in one half of patients (*3*), childhood obesity or excess weight in the majority (*4*), and a 8× higher prevalence of type 1 diabetes than in an age-matched control population (*5*). In addition, specific alterations in immune response are frequently present, including leukopenia, chemotactic defects (*6*), decreased immunoglobulin G_4_ levels (*7*), and T- and B-cell abnormalities (*7**,**8*) with reduced B lymphocytes (*9*). Patients with Down syndrome have an increased need for hospitalization because of lower respiratory tract disease caused by respiratory syncytial virus (*10*) and other respiratory infections (*11*) as well as reduced access to healthcare (*12*). An increased proportion of patients with Down syndrome have pneumonia as cause of death (*13*). However, respiratory infections have not been associated with congenital heart disease (*14*). Although persons with Down syndrome are likely at increased risk for complications, these patients are not explicitly listed in groups that should receive priority vaccination or for early treatment of influenza. The objective of the study was to determine whether Down syndrome was associated with adverse outcomes in cases of influenza-like illness (ILI) and severe acute respiratory illness (SARI) during the first months of the outbreak of influenza A pandemic (H1N1) 2009 virus (*15*).

## The Study

In the ILI/SARI database of the Mexican Ministry of Health, we identified all persons for whom Down syndrome (World Health Organization’s International Statistical Classification of Disease and Health-related Problems, 10th revision [ICD-10] codes Q-90.0 to Q-90.9) was cited among their coexisting conditions or among the causes of death from May 2009 through October 2009. In Mexico, influenza is a disease of mandatory notification, and the database includes all cases reported from 597 sentinel health units, including outpatient clinics as well as hospitals from all 32 states. Notification includes obtaining information for each patient on such factors as hospitalization, antiviral drug treatment, and complications, and we compared selected characteristics of patients with and without Down syndrome. ILI is defined as fever (>38ºC) with cough and headache and 1 additional respiratory or digestive symptom. The definition of SARI adds difficulty breathing or acute respiratory failure to the ILI definition.

We also reviewed the hospital database from the National Institute of Respiratory Diseases (INER) for associations among Down syndrome, hospitalizations, and adverse outcomes. INER is a national referral center for respiratory diseases that cares mainly for patients from metropolitan Mexico City who lack health insurance. For analysis, we included all patients with discharge diagnoses of pneumonia and influenza (ICD-10 codes J09–J18).

We compared the prevalence of hospitalization, intubation, and death among patients with Down syndrome and among the remaining patients. As estimators of association, we used odds ratios (ORs) with 95% confidence intervals (CIs), and to adjust for additional variables such as age or sex, we used logistic regression. We also described medians and interquartile ranges (IQRs) for selected variables and compared age and number of coexisting conditions by the standard *t* test for independent groups.

The ILI/SARI database had 214,902 reported cases during the pandemic; mean patient age was 23 years (SD 17 years); 52% of these patients were female, 83% were outpatients, 9% were hospitalized, and the remaining 8% did not have a record of hospitalization. Among the reported cases, 45,772 patients had a record of coexisting conditions, mainly diabetes and asthma. We found 60 (0.03%) patients with reported ILI/SARI who also had a diagnosis of Down syndrome. Patients with and without Down syndrome had a similar median time from onset of signs and symptoms to hospitalization (2 days, IQR 1–4 days), and the frequency of symptoms reported was similar.

Treatment with oseltamivir was provided for most hospitalized patients (for 92.0% with Down syndrome and 85.2% without Down syndrome). Persons with Down syndrome were younger on average than the remainder of patients (median age 8.5 years, IQR 3–26 years, vs. 20 years, IQR 9–34 years, respectively). In Mexico, seasonal influenza vaccination is recommended for children <3 years of age, but only 24% of patients with Down syndrome within this age range were reported to have received the vaccine, compared with 33% patients without Down syndrome (OR 0.5, 95% CI 0.1–2.4, p>0.05).

According to the ILI/SARI database, patients with Down syndrome had an increased risk for hospitalization, endotracheal intubation, and death compared with patients without Down syndrome. Hospitalization was reported for 61.7% of patients with Down syndrome compared with 9.2% of patients without Down syndrome (crude OR 15.9, 95% CI 9.5–26.8; age-adjusted OR 21.2, 95% CI 12.4–36.4). Endotracheal intubation of patients for whom information was available was reported for 18.2% of case-patients with Down syndrome and 2.6% of those without Down syndrome (crude OR 8.2, 95% CI 3.4–19.9); 23.3% of those with Down syndrome died vs. 0.1% of those without Down syndrome (crude OR 335, 95% CI 181–619, age-adjusted OR 521, 95% CI, 274–991).

From January 2000 through June 25, 2009, a total of 42,298 admissions to INER were registered; patients had a mean age of 42 years, (SD 23 years), and 53% of these were male. Fifty-nine patients had a diagnosis of Down syndrome (0.14%), and 12 of these had a diagnosis of pneumonia or influenza (20.3%) vs. 6.8% of the remaining population (OR 3.5, 95% CI 1.7–6.5, p<0.05). Patients with Down syndrome had an increased risk for in-hospital death (age- and gender-adjusted OR 4.6, 95% CI 2.1–9.7, p<0.05). Although patients with Down syndrome were younger (mean age 15.2 years vs. 41.6 years, p<0.001), they were more likely to have a disease of the cardiovascular system (29% vs. 16%, OR 2.1 95% CI 1.2–3.7, p = 0.01), a congenital malformation of the cardiovascular system (19% vs. 0.5%, OR 47, 95% CI 24–93, p<0.001), and more coexisting conditions per patient (3.3 from 4 possible vs. 2.1, p<0.001).

## Conclusions

Persons with Down syndrome often manifest a variety of immune defects and several risk factors for adverse outcomes for pandemic (H1N1) 2009, including obesity, diabetes, and cardiovascular diseases. Down syndrome patients are not explicitly listed in the groups of patients at increased risk for influenza, so early antiviral drug treatment and priority vaccination are prescribed for Down syndrome patients only if a high-risk health condition is found, for example, a cardiovascular disease or if persons with Down syndrome are considered to have a cognitive disorder (*2*). These factors may lead to inconsistent healthcare for those affected with Down syndrome and to missed opportunities for prevention and early treatment of ILI.

Patients with Down syndrome and ILI/SARI reported to the Mexican Ministry of Health had an increased risk for hospitalization, endotracheal intubation, and death. Consistent with the national database, in a referral respiratory center, patients with Down syndrome were more likely to have a diagnosis of pneumonia or influenza and to die during hospitalization.

The Ministry of Health database lacks detailed clinical information, and surveillance is based on sentinel health units chosen to be representative of the whole Mexican health system and that would provide information of good quality. The main risk factors for influenza are routinely requested and reported. However, the INER database includes diagnoses after hospital discharge but solely from 1 referral hospital. Yet both sources confirmed an increased risk for hospitalization and death for patients with Down syndrome and ILI. Patients with Down syndrome should be vaccinated against the seasonal influenza viruses and the influenza A pandemic (H1N1) 2009 virus. Early treatment of Down syndrome patients for ILI should be promoted by health systems and Down syndrome organizations.
